# COVID-19 risk perceptions and precautions among the elderly: A study of CALD adults in South Australia

**DOI:** 10.12688/f1000research.74631.1

**Published:** 2022-01-13

**Authors:** Mohammad Hamiduzzaman, Noore Siddiquee, Helen McLaren

**Affiliations:** 1 College of Health, Medicine & Wellbeing, The University of Newcastle, Department of Rural Health, Taree, New South Wales, 2430, Australia; 2 College of Business, Government & Law, Flinders University, Adelaide, South Australia, Australia; 3 College of Education, Psychology and Social Work, Flinders University, Adelaide, South Australia, Australia

**Keywords:** Culturally and linguistically diverse community, older adults, risk perceptions, health precautions, behavioural dimensions, emergency preparedness, South Australia

## Abstract

**Background:** Coping with COVID-19 is a challenge for culturally and linguistically diverse (CALD) older adults. In Australia, little attention has been given to understanding associations between cultural contexts, health promotion, and socio-emotional and mental health challenges of older CALD adults during the COVID-19 pandemic. Therefore, we have collected data from older CALD adults to examine their COVID-19 risk perceptions and its association with their health precautions, behavioural dimensions and emergency preparation.

**Methods: **A cross-sectional survey was conducted in South Australia. The CALD population aged 60 years and above were approached through 11 South Australian multicultural NGOs.

**Results:** We provide the details of 155 older CALD South Australians’ demographics, risk perceptions, health precautions (problem-and-emotion-focused), behavioural dimensions and emergency preparation.  The explanatory variables included demographic characteristics (age, gender, education and ethnicity); and risk perception (cognitive [likelihood of being affected] and affective dimension [fear and general concerns], and psychometric paradigm [severity, controllability, and personal impact]. The outcome measure variables were health precautions (problem-focused and emotion-focused), behavioral adaptions and emergency preparation.

**Conclusions:** This dataset may help the researchers who investigate multicultural health or aged care in the pandemic and or who may have interest to link with other datasets and secondary use of this primary dataset in order to develop culturally tailored pandemic-related response plan. The data set is available from Harvard Dataverse.

## Introduction

The COVID-19 pandemic is a global and national health emergency, affecting many Australians’ everyday lives and livelihood, including older adults. ^1^ Older Australians aged 65 years and above account for 15% of the country’s total population, of which three in 10 were born overseas. ^2^ The relationships between older Australian’s cultural backgrounds, health and quarantine protocols, as well as psychosocial and emotional wellbeing during the pandemic are unclear across the country, including in South Australia.

The pandemic has strained hospitals, public health and social services organizations in South Australia, as in other Australian jurisdictions, which raises concerns among older CALD adults about their health and emotional wellbeing. Older adults are more likely to be infected and die (i.e., about 78% of all COVID-19 patients who died were aged 65 years and above) than other age groups. ^3^^,^^4^ The risks and fear are higher for older CALD adults because of common health concerns, such as a high rate of chronic conditions and comorbidities, avoiding medical appointments and acute care, chance of delaying care; risk of community transmission due to reluctance; and prevalence of depression and anxiety because of a separation from families and friends and a restriction of movement. ^5^^,^^6^ They have been anticipated to experience changes in their wellbeing and quality of life during the pandemic, which have not been investigated. In addition, COVID-19 risk perceptions, psychological and emotional health-related worries and coping behaviours may vary in older CALD adults based on their age, gender, education, and ethnicity. Therefore, we have collected data from older CALD adults to examine their COVID-19 risk perceptions and their association with health precautions, behavioural adaptations and emergency preparation based on socio-demographic characteristics.

## Methods

This data note presents data from a cross-sectional study in South Australia from July 1 to December 31, 2020, involving basic demographic data collection and assessment of risk perceptions, health precautions, and emergency preparedness, to understand the relationships between risks perceptions of older CALD adults with their problem- and emotion-focused precautions, behavioural coping and emergency preparedness during the COVID-19 pandemic.

### Source of data

Data were collected from older CALD adults (≥60 years) through South Australian multicultural NGOs. The multicultural NGO selection process involved a primary search in the list of Australian accredited NGOs. Fifteen NGOs in South Australia were deemed appropriate for three reasons: they were accredited and active in the community; represented the majority of CALD communities; and had members of older CALD adults. The online survey (SurveyGizmo) was administered by sending an email invitation to 11 South Australian multicultural NGOs, who agreed to voluntarily distribute the participation information sheet and survey link to their older community members, resulted in 155 surveys completion. According to the Australia Bureau of Statistics, approximately 95,000 older CALD adults lived in South Australia in 2018. In our study, we aimed to approach 300 respondents based on the number of multicultural NGOs having agreed to support this study and computer illiteracy rates among the older CALD adults, which allowed for a dropout rate of less than 50%. We continued to collect data over a period of six months until we reached the saturation.

The outcome measures were health precautions (problem-focused and emotion-focused), behavioural dimensions and emergency preparation for coping with the COVID-19 crisis. The scale included nine items relating to problem-focused precautions and 10 items assessing emotional precautions. Seven behavioural dimensions items and five related to emergency preparedness during the pandemic were considered as outcome measures. The demographic characteristics and risk perception data were collected as explanatory variables. Participant demographics were categorized into: age (60-69 years, 70-79 years, and 80 years and above); gender (male and female); education (no formal education, primary school, high school, Bachelors, and Masters and above); and ethnicity (country of birth; classified as Asian, African, and non-English speaking self-nominated CALD European). We used the modified version (i.e., a 15-indicator risk perception scale) of Gerhold’s (2020) COVID-19 risk perception measure, which was developed based on Slovic’s (1987) psychometric concepts— a) cognitive (i.e., likelihood of being affected) and affective dimension (i.e., fear and general concerns), and b) psychometric paradigm (i.e., severity, controllability, and personal impact).

Descriptive analysis was conducted for demographics, indicators of risk perceptions, as well as the problem- and emotion-focused precautions, behavioural dimensions and emergency preparation. To examine the associations between the explanatory variables and the outcome measures, several multiple linear regression models were fitted. Multicollinearity was checked in the regression analyses by examining the tolerance values. The entire analysis was performed with STATA/MP version 13.0 (StataCorp, LP, College Station, Texas, USA).

### Dataset validation

This dataset has some limitations. It is a cross-sectional study, therefore, causal inferences about the relationship between socio-demographic characteristics and risk perceptions, health precautions, behavioural adaptations, and emergency preparation could not be measured. Our study sample was not representative of all CALD communities in South Australia and the findings may not be generalised to other CALD populations, either in Australia or overseas. Also, we relied on an English-language online questionnaire for data collection, which had an influence on participation rates.

However, we employed several strategies to ensure validity and reliability of the dataset. In measuring the risk perceptions, we used Gerhold’s (2020) COVID-19 risk perception scale, developed based on Slovic’s (1987) psychometric concepts: a) cognitive (i.e., likelihood of being affected) and affective dimension (i.e., fear and general concerns); and b) psychometric paradigm (i.e., severity, controllability, and personal impact). The 19 indicators of health precautions, seven items of behavioural adaptations, and five items of emergency preparation scales were drawn from Folkman and Lazarus’s (1988) problem- and emotion-focused coping, which have been used by scholars to measure similar outcome variables in other countries. Reliability in data items was separately checked using Cronbach’s α, a commonly cited test to check internal consistency of a scale. ^7^ Internal consistencies were: three items of becoming infected (α = 0.9073); two-items of fear (α = 0.8863); nine items of problem-focused health precautions (α = 0.7964); 10 items of emotion-induced health precautions (α = 0.7050); seven items of behavioural adaptations (α = 0.7927); and five items emergency preparations was 0.83 (Cronbach’s α = 0.8292).

In order to control bias, the SurveyGizmo software was used for data collection. The participants were approached and recruited by the NGOs, and they were given the opportunity to share their email address to receive a copy of study results and review.

### Ethical approval

The study received ethical approval from the Flinders University Human Research Ethics Committee, Australia (Approval number: HEL2215). A group of 11 South Australian multicultural NGOs provided written informed consent in supporting the researchers for participant approach and recruitment. Informed consent for the participation was implied, as the completion of online survey was considered written informed consent. The data collected from participants were only used for this research.

## Data availability

Harvard Dataverse: *COVID-19 Risk Perceptions and Precautions among the Elderly: A Study of CALD Adults in South Australia,* https://doi.org/10.7910/DVN/OUGSUC. ^8^

This project contains the following underlying data:

• COVID-19 Latest Dataset.SPSS. (Contains data in SPSS of the older CALD adults’ socio-demographic variable, risk perceptions, problem and emotion-focused precautions, behavioral dimensions and emergency preparation).
