# Supplementary material for: COVID-19 risk perceptions and precautions among the elderly: A study of CALD adults in South Australia
Source: F1000Res. 2022 Jan 13;11:43. [Version 1] doi: 10.12688/f1000research.74631.1 (PMC8933644; doi:10.12688/f1000research.74631.1)
Supplement: Peer review history

# Peer review: Reviewer response for version 1

**DOI:** 10.5256/f1000research.78406.r125331

**Published:** 2022-03-18

**Authors:** Sharif Rasel

**Affiliations:** 1 College of Business, Government and Law, Flinders University, Adelaide, SA, Australia

Thanks for the opportunity to review this article. The authors have followed a rigorous statistical method to conduct the data analysis. However, the authors may consider to report the test of common method variance and non-response bias. Further, they may also include factor loadings of the items. In my view, the article may be considered for publication.

Are sufficient details of methods and materials provided to allow replication by others?

Yes

Is the rationale for creating the dataset(s) clearly described?

Yes

Are the datasets clearly presented in a useable and accessible format?

Yes

Are the protocols appropriate and is the work technically sound?

Yes

Reviewer Expertise:

business research involving quantitative analysis

I confirm that I have read this submission and believe that I have an appropriate level of expertise to confirm that it is of an acceptable scientific standard.

# Peer review: Reviewer response for version 1

**DOI:** 10.5256/f1000research.78406.r123067

**Published:** 2022-03-07

**Authors:** Binahayati Rusyidi

**Affiliations:** 1 Padjadjaran University, Sumedang, Indonesia

The title of this study is interesting. The research is also important because the issues are understudied.

 However, in my opinion, there is an inconsistency between the purpose of the study and the results. Based on the research questions, I expected to get comprehensive descriptions and analysis about factors associated with Covid-19 risk perceptions among CALD adults. The manuscript, unfortunately, provides little information about the results and discussion of the findings.

 If the study is to report the results from a study, I suggest the authors do some revisions that follow the standard for publishing an empirical study.

 In addition, some additional revision or clarification could be incorporated including:

 a. Strengthening the position of the study within the existing knowledge about the issue. While the authors have stated that the topic covered by their research is rarely studied in Australia, it is important to see what is available globally.

 b. It will be better if the authors can provide a socio-demographic description of 95,000 CALD adults in South Australia in 2018. This information can be helpful when doing the analysis of the study, for example to check whether the participants of the study proportionately or disproportionately represent the CALD adults general population.

 c. It might be more helpful for the readers if the authors can provide examples of items assessed by the instruments used to measure dependent and independent variables.

 d. The information for validity and reliability checking of Covid-19 risk perception measure can be moved to source of data sub-section. There is limited information about  instruments used to measure outcome variables, so I assume they are self-developed instruments? Information about internal consistency tests for these instruments can be provided as well.

 e. I am wondering why the length of stay in Australia and the nature of living arrangement of participants are not assessed as demographic variables in the study because I think they are possibly associated with the outcome variables. Some explanation about this?

 f. The term "saturation" stated in the data collection process is usually used in qualitative research, not in quantitative study.

 g. I am not clear with the term computer illiteracy rates stated in the manuscript. Did the researchers try to explain that they anticipated high rates of CALD adults who are computer illiterate?

 h. The researchers mentioned multicollinearity checking but there was no information whether multicollinearity was an issue or not.

Are sufficient details of methods and materials provided to allow replication by others?

Partly

Is the rationale for creating the dataset(s) clearly described?

Partly

Are the datasets clearly presented in a useable and accessible format?

Yes

Are the protocols appropriate and is the work technically sound?

Partly

Reviewer Expertise:

qualitative research; social welfare

I confirm that I have read this submission and believe that I have an appropriate level of expertise to confirm that it is of an acceptable scientific standard, however I have significant reservations, as outlined above.

# Peer review: Reviewer response for version 1

**DOI:** 10.5256/f1000research.78406.r119644

**Published:** 2022-01-19

**Authors:** Dr. Yang Wang

**Affiliations:** 1 Zilber School of Public Health, University of Wisconsin Milwaukee, Milwaukee, WI, USA

Thanks for inviting me to peer review this paper.

 This paper reported a survey to older CALD adults about risk perceptions and precautions in South Australia. However, I still cannot understand its aim very well, because there is a contradiction between its title, research question and results: is the aim of this paper to report the finding of a cross-sectional survey or just provide a dataset?

 If its aim is to provide a dataset, it is proper to be published after revision, because it can help more people to understand the situation of older CALD adults under the threat of covid-19. If its aim is to report the finding of a cross-sectional study, the authors may need to provide more information in the manuscript according to STROBE checklist.

 Specifically, I suggest the authors should provide more information to readers more clearly:

 (1) How many jurisdictions are there in Australia? Is the situation of older CALD adults between South Australia and other jurisdictions similar?

 (2) What are the inclusion and exclusion criteria for select the participants (older CALD adults)?

 (3) References 5 and 6 do not provide supportive information about the higher risk of older CALD adults. Please check it.

 (4) For a cross-sectional survey, saturation is not an applicable concept (it is often used in qualitative research). What we need to know is the instrument, survey approach, sample size and sampling method, because they can decide if we can obtain reliable information representing the real situation of the target population.

 (5) What are “computer illiteracy rates”? I can't understand its relationship with older CALD adults.

 (6) The information about the verification of Gerhold’s COVID-19 risk perception scale, Folkman and Lazarus’s problem and emotion-focused coping may need to be moved to the 'Source of data' section, because it is the verification of the instrument. The validation of the dataset may be different to the validation of the instrument. Please read: https://www.ncbi.nlm.nih.gov/pmc/articles/PMC6395976/

 (7) If the aim of this paper is only to provide a dataset, it seems unnecessary to use multiple linear regression to calculate the causal relationship between factors.

 Best wishes

Are sufficient details of methods and materials provided to allow replication by others?

Partly

Is the rationale for creating the dataset(s) clearly described?

Yes

Are the datasets clearly presented in a useable and accessible format?

Yes

Are the protocols appropriate and is the work technically sound?

Partly

Reviewer Expertise:

Science of science and research method for primary care research

I confirm that I have read this submission and believe that I have an appropriate level of expertise to confirm that it is of an acceptable scientific standard, however I have significant reservations, as outlined above.
